# Interleukin-4 Promotes Myogenesis and Boosts Myocyte Insulin Efficacy

**DOI:** 10.1155/2019/4182015

**Published:** 2019-11-11

**Authors:** Yih-Hsin Chang, Jen-Ning Tsai, Tzu-Lin Chen, Kuo-Ting Ho, Hsin-Yi Cheng, Chiao-Wan Hsiao, Ming-Yuh Shiau

**Affiliations:** ^1^Department of Biotechnology and Laboratory Science in Medicine, National Yang-Ming University, Taipei, Taiwan; ^2^Program in Molecular Medicine, National Yang-Ming University and Academia Sinica, Taipei, Taiwan; ^3^Department of Medical Laboratory and Biotechnology, Chung Shan Medical University, Taichung, Taiwan; ^4^Clinical Laboratory, Chung Shan Medical University Hospital, Taichung, Taiwan; ^5^Department of Nursing, College of Nursing, Hungkuang University, Taichung, Taiwan

## Abstract

Anti-inflammatory cytokine interleukin-4 (IL-4) promotes glucose tolerance and insulin sensitivity while reduces lipid deposits. However, the effects of IL-4 on energy metabolism in muscle, the largest insulin-targeting organ, remain obscure. The study aimed at addressing the roles of IL-4 in myocyte differentiation (myogenesis) and energy metabolism of muscle cells. Effects of IL-4 on myogenesis, and interaction between IL-4 and insulin on glucose metabolism of C2C12 myoblasts and the terminal differentiated myocytes were analyzed. IL-4 improved GLUT4 translocation and tended to elevate glucose uptake by boosting insulin signaling. In diabetic mice, transient and long-term IL-4 showed differential effects on insulin signaling and efficacy. The study provides evidence to address the roles of IL-4 in mediating whole-body muscle reservoir and glucose metabolism, as well as the interaction between immune responses and energy homeostasis. IL-4 has dual potential to act as an adjuvant therapeutic target for sarcopenia to preserve muscle mass and insulin resistance to improve insulin sensitivity, which implicates the regulation of immune system to the muscle differentiation and exercise performance.

## 1. Introduction

Type 2 diabetes mellitus (T2DM) is a common endocrine disease characterized by hyperglycemia and insulin resistance [1]. Hotamisligil et al. first demonstrated that expression of tumor necrosis factor-*α* (TNF-*α*) in adipose tissue of obese animals is markedly increased [2]. From then on, accumulating studies proved that T2DM is an inflammatory condition with elevated acute phase inflammatory reactants [3–6]. Excess glucose and nutrient intake leads to oxidative stress which induces elevated levels of pro-inflammatory cytokines. These increased cytokines, such as interleukin-6 (IL-6), result in impaired insulin action on peripheral glucose metabolism [7, 8]. Therefore, cytokines are involved in the development of insulin resistance. While the role of Th2-derived cytokine IL-6 is extensively studied in glucose metabolism, much less regarding putative participation of other Th2 cytokines in insulin efficacy and metabolic homeostasis is uncovered. Accordingly, it is intriguing for us to explore if other Th2 cytokines are also involved in glucose homeostasis.

Anti-inflammatory interleukin-4 (IL-4) executes pleiotropic immune-regulatory functions [9]. According to our previous reports, significant association between *IL-4* genotypes with T2DM and circulatory high density lipoprotein-cholesterol (HDL-C) [10], as well as between IL-4 receptor alpha chain (IL-4R*α*) genetic polymorphisms with HDL [11], is identified. IL-4 improves insulin sensitivity and glucose tolerance while inhibits lipid accumulation [12, 13] through lipolysis-promoting and adipogenesis-inhibiting capacity [14, 15]. The above results not only uncover novel function of IL-4 in regulating glucose/lipid metabolism, but reveal the involvement of IL-4 in metabolic abnormalities such as obesity and T2DM.

Adipose tissue dysfunctions are associated with decreased glucose uptake and mitochondrial oxidative phosphorylation in muscle cells [16]. IL-4 is known to promote insulin signaling in adipocytes [17]. Recently, we demonstrate that IL-4 boosts insulin-induced energy deposits by enhancing glucose uptake and lipogenesis in hepatocytes [18]. While IL-4 is reported as a myoblast recruitment factor which promotes muscle differentiation (myogenesis) and regeneration [19, 20], the effects of IL-4 on energy metabolism in muscle, the largest insulin-targeting organ, remain obscure. Therefore, regulation of IL-4 to myogenesis and glucose metabolism in the undifferentiated C2C12 myoblasts and terminal differentiated myocytes were examined in the present study. The influences of IL-4 to Akt and glycogen synthase kinase-3*β* (GSK-3*β*) in muscle cells under diabetic or insulin resistant conditions were also examined *in vivo*. Our results demonstrate that IL-4 promotes myogenesis by up-regulating the expression of myocyte-specific markers, transcription factors and kinases required for different stages of myogenesis. Additionally, IL-4 improves translocation of glucose transporter 4 (GLUT4) and tends to elevate glucose uptake ability by boosting insulin signaling in both myoblasts and myocytes.

## 2. Materials and Methods

### 2.1. Materials

Antibodies against Akt, phospho-Akt (p-Akt) Ser473, cyclin-dependent kinase 5 (CDK5), GSK-3*β*, and p-GSK-3*β* Ser9 were from Cell Signaling Technology (Danvers, MA, USA); anti-IL-4R and anti-GLUT4 from Abcam (San Francisco, CA, USA); anti-myogenin from Millipore (Temecula, CA, USA), anti-myogenic factor 6 (MYF6) from Taiclone Biotech Corp. (Taipei, Taiwan); ECL reagent from Calbiochem (Merck Millipore, Billerica, MA, USA); insulin, anti-GATA-binding protein 3 (GATA3), anti-*β*-actin from Sigma (St. Louis, MO, USA); Trizol Reagent from Life Technology (Carlsbad, CA, USA); anti-myosin heavy chain (MyHC) from SantaCruz; IL-4 ELISA kit from R&D systems, Inc. (Minneapolis, MN, USA).

### 2.2. Cell Culture System

C2C12 myoblasts were maintained in DMEM containing 10% FBS and 1% penicillin-streptomycin in 5% CO_2_ at 37°C. Cells were cultured in DMEM containing 3% horse serum (HS) for 6 days to render the cells differentiate into mature myocytes. Myotube formation was determined as the presence of multinuclei-containing tubing morphology. Markers of mature myocytes and transcription factors/kinases required for different stages during myogenesis, including muscle-restricted coiled-coil protein (MURC), myogenin, CKD5, MYF6 and MyHC were analyzed by RT-PCR and/or Western blotting. For *in vitro* IL-4 and/or insulin treatment, cells were exposed to IL-4 (10 ng/mL) and/or insulin (100 nmol/L) for the time indicated.

### 2.3. Fluorescence Microscopy

Cells were fixed with 3.7% formaldehyde for 15 min and then permeabilized with 0.5% triton X-100 for 10 min at room temperature. The cells were then blocked with 5% BSA for 30 min, incubated with fluorescent dye-conjugated actin (Invitrogen) for 1 h at room temperature, followed by mounted with GEL/MOUNT containing DAPI (Molecular Probes, Eugene, USA). The images were taken by ZEISS LSM 700 confocal fluorescence microscope using 63X objective len.

### 2.4. RT-PCR

Total cellular RNA was extracted using Trizol Reagents. Complementary DNA was synthesized using total RNA (5 *μ*g), oligo dT primer (200 pmol), and 5× MMLV RT. The synthesized cDNA was amplified in 30 *μ*L reaction mixtures using target sequence-specific PCR primers as listed in Table 1. All RT-PCR reactions were carried out with initial 95°C denaturation for 5 min and the following thermocycles: 95°C for 1 min, annealing for 1 min and 72°C for 1 min. PCR products were separated by electrophoresis and visualized using an ultraviolet transilluminator.

### 2.5. Western Blot

Total proteins were extracted from cells at 4°C by lysis buffer (50 mmol/L Tris, pH 7.5, 150 mmol/L NaCl, 1% Triton, 2 mmol/L EDTA, 1 mmol/L Na_3_VO_4_) containing protein inhibitors. For animal studies, protein extracts from quadriceps muscle were obtained by homogenizing the tissue with T-PER reagent (Pierce, Rockford, IL, USA) supplied with phosphatase and protease inhibitors (Roche, Indianapolis, IN, USA). Protein extract samples were resolved by SDS-PAGE and electrotransferred to PVDF membrane. Membranes were permeated in TBST buffer (150 mmol/L NaCl, 50 mmol/L Tris, pH 7.4, 0.1% Tween 20) with 5% milk for 1 h, incubated with primary antibodies at 4°C overnight, followed by secondary antibodies (ZYMED Laboratories Inc & NEN, Boston, USA) for 1 h. After washing, proteins were visualized and quantitated using ECL reagents (Millipore Corporation).

### 2.6. GLUT4 Translocation

Cells were treated with IL-4 and/or insulin for 30 min after 3 h serum starvation, fixed with 4% paraformaldehyde and incubated with anti-GLUT4 for 1 hr at 4°C, followed by adding secondary antibodies (Millipore) after washing. Then o-phenylenediamine dihydrochloride (OPD)-containing phosphate-citrate buffer (0.05 M) was added, and absorbance at 450 nm was measured and quantitated (Infinite 200).

### 2.7. Glucose Uptake

Glucose uptake assay was performed after cells were incubated with glucose-free KRPH buffer (118 mmol/L NaCl, 5 mmol/L KCl, 1.3 mmol/L CaCl_2_, 1.2 mmol/L MgSO_4_, 1.2 mmol/L KH_2_PO_4_, and 30 mmol/L HEPES, pH 7.4) for 3 h. Cells were treated with IL-4 and/or insulin for 20 min, fed with 100 *μ*mol/L 2-[N-(7-nitrobenz-2-oxa-1,3-diazol-4-yl)amino]-2-deoxy-D-glucose (2-NBDG) for 10 min, followed by adding ice-cold KRPH buffer containing 10 mM glucose. Cells were washed with ice-cold PBS and lysed, and intracellular fluorescence intensity was measured (485/540 nm, Infinite 200).

### 2.8. Enzyme-Linked Immunosorbent Assay

Cell-free supernatants were harvested on the day indicated during myogenesis, and IL-4 levels were analyzed by IL-4 ELISA kit according to the manufacturer's instructions.

### 2.9. Animal Experiments

For adenovirus experiments, 8-week-old male C57BL/6 mice were *i.p.* injected once daily for consecutive 2 days (2 times in total) with 5x10^11^ adenovirus containing IL-4-coding gene (AdIL-4) or AdLacZ, followed by *i.p.* streptozotocin (STZ; 100 mg/kg; Sigma-Aldrich, St Louis, MO, USA) administration on the second day to induce diabetic onset as described [12]. For high fat diet (HFD) experiments, 4-week-old male C57BL/6 mice were randomly grouped and fed with HFD (D12492, Rodent Diet with 60 kcal% fat) to induce insulin resistance or standard chow diet (SD; LabDiet 5010 with 13 kcal% fat) purchased from Research Diet Inc. (NJ 08901 USA), and *i.p.* administered with recombinant IL-4 (1,000 pg per mouse; BD Pharmingen) every other day for 8 weeks. Insulin resistant status of HFD mice was confirmed as described [12]. Protein extracts from quadriceps muscle were obtained, respectively, after the harvested tissues were homogenized by T-PER tissue protein extraction reagent (Pierce, Rockford, IL, USA) supplied with phosphatase and protease inhibitors (Roche, Indianapolis, IN, USA) after the mice were injected with insulin (0.15 IU/g body weight). For examining insulin signaling, p-Akt and p-GSK3*β* were analyzed by Western blot. Animal protocols were reviewed and approved by the Institutional Animal Care and Use Committee, National Yang-Ming University, and Chung Shan Medical University animal studies committee, with all methods performed in accordance with the relevant guidelines and regulations.

### 2.10. Statistical Analysis

Each experiment was carried out at least three times. Results were presented as mean ± SEM. Significant difference between groups was analyzed by two-tailed unpaired Student *t*-test or one-way ANOVA. Statistical difference was defined as *p* < 0.05.

## 3. Results

### 3.1. C2C12 Myocyte Differentiation

**Figure 1:**
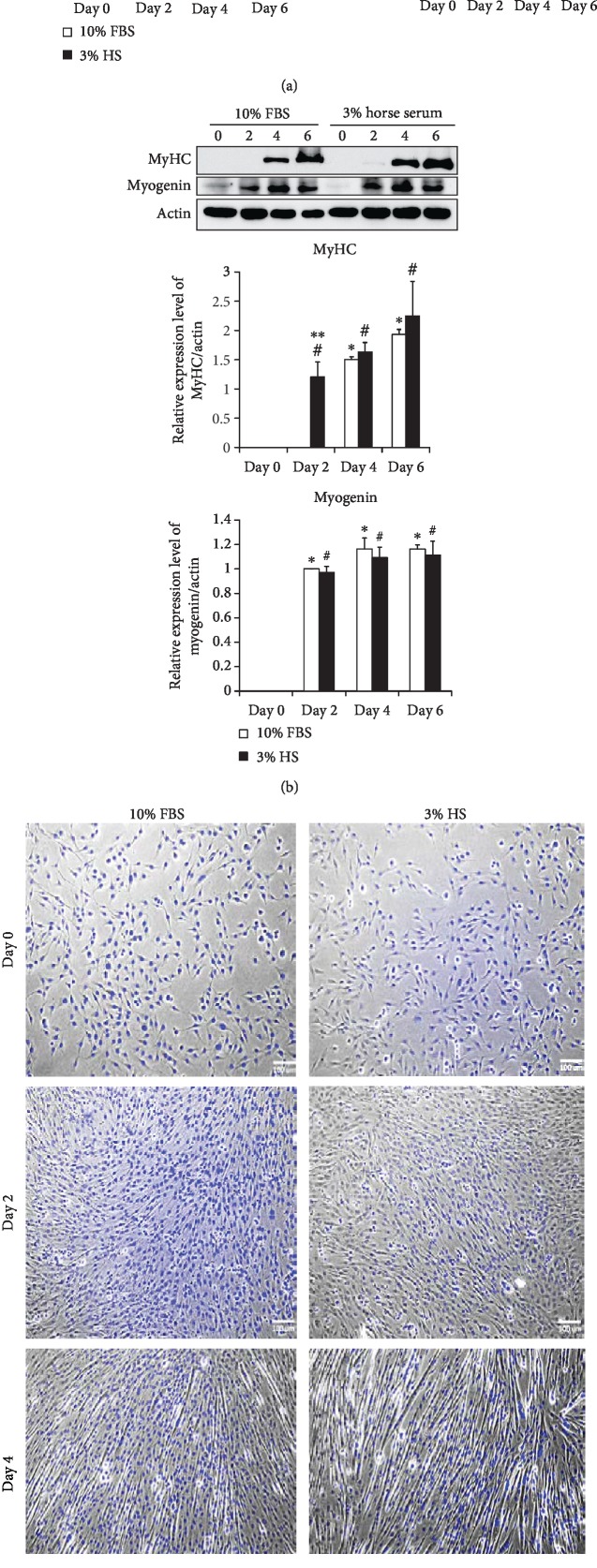
C2C12 myogenic differentiation. Expression of MURC, myogenin and MyHC mRNA (a) and protein (b) from C2C12 cells at the time indicated during myogenesis in media containing either 10% fetal bovine serum (FBS) or 3% horse serum (HS) was examined (n = 3). ^∗^p < 0.05 and ^∗∗^p < 0.01 v.s 10% FBS at day 0, ^#^p < 0.05 v.s 3% HS at day 0, ^∫^p < 0.05 v.s 10% FBS group at the same day. (c) Morphology of cells at the indicated day was stained with DAPI and examined by fluorescence microscopy.

C2C12 myoblasts were allowed to differentiate in culture media containing 10% FBS or 3% HS. The mRNA (Figure 1(a)) and protein (Figure 1(b)) levels of myocyte markers, including MURC, myogenin and MyHC were temporally examined to analyze the extent of differentiation. Presence of multinucleated cells was observed on day 4 (Figure 1(c)). Mature myotubes in both condition aligned in parallel direction on day 6, however, cells in 3% HS contained more nuclei in average (3-5/cell) than their counterparts (3-4/cell). The above results indicate that although C2C12 cells differentiate well in both condition, 3% HS is a better environment for myocyte differentiation. Therefore, 3% HS is used as the myogenesis-inducing condition for the following study.

### 3.2. IL-4 Promotes Myogenesis

**Figure 2:**
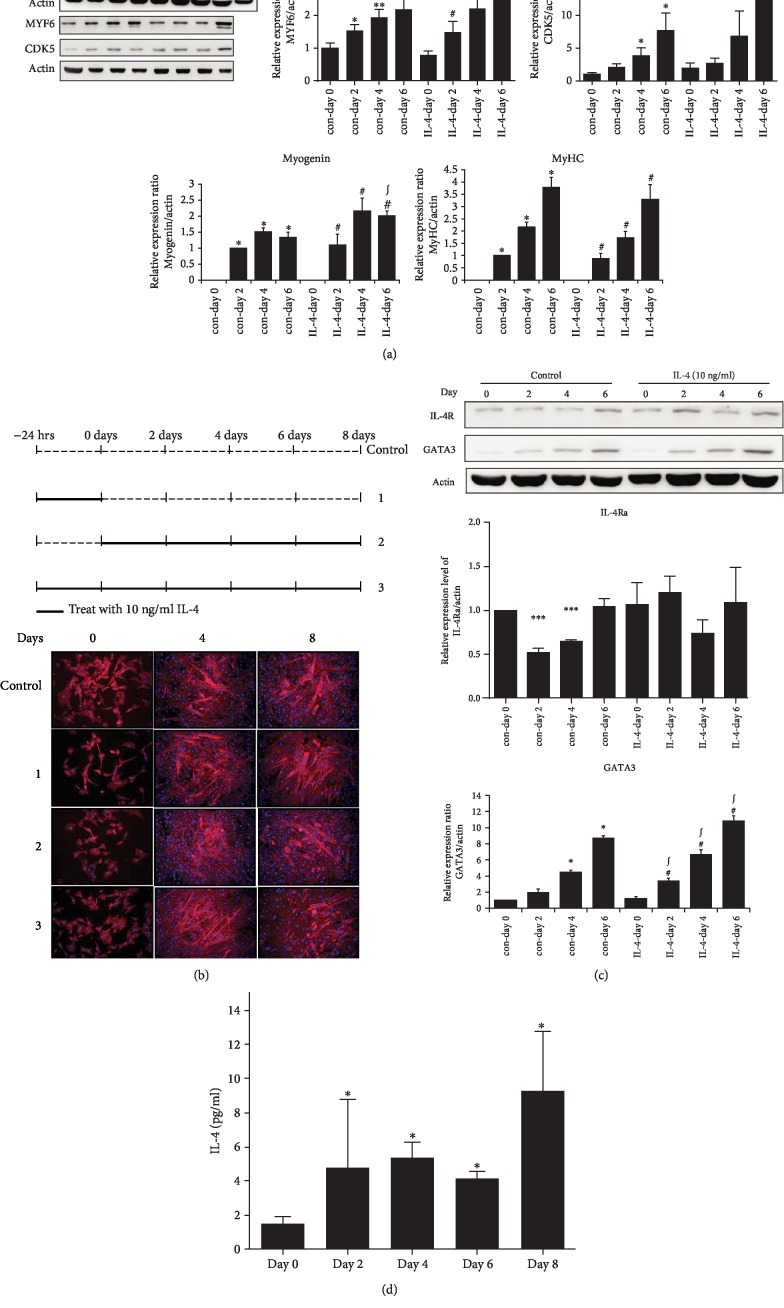
IL-4 promotes myogenesis by up-regulating expression of myocyte-specific marker. (a) Levels of myogenin, MYF6, CDK5 and MyHC protein expression at the time indicated were analyzed. (b) Morphology of C2C12 cells at the indicated day. (c) Cell lysates were harvested at the time indicated and IL-4R and GATA3 was analyzed (n = 3). ^∗^p < 0.05 and ^∗∗^p < 0.01 v.s control at day 0, ^#^p < 0.05 v.s IL-4 at day 0, ^∫^p < 0.05 v.s control at the same day. (d) IL-4 in culture medium harvested at the day indicated was measured by ELISA. ^∗^*p* < 0.05 v.s control at day 0.

Extent of differentiation was temporally examined in cells differentiated under various schemes of IL-4 treatment. Although there was no significant difference of MyHC expression in the presence or absence of IL-4 exposure, myogenin was significantly promoted by IL-4 (Figure 2(a)). In addition, expression of muscle cell hypertrophy- required transcription factor MYF6 and myogenesis- promoting and establishing CDK5 [21] were also up-regulated by IL-4. The morphology of cells differentiated in the environment with differential IL-4 treatment scheme (as depicted in Figure 2(b)) was further analyzed to examine the myogenesis-promoting capacity of IL-4. The differentiated mature myotubes under IL-4 exposure contained more nuclei (8-13/myotube) and were thicker with longer diameter on day 8 (Figure 2(b)). It indicates that IL-4 promotes myogenesis by enhancing the expression of myocyte marker genes, as well as myogenesis-promoting transcription factors and kinases at different stages.

The expression of IL-4R and IL-4 downstream target gene GATA3 in the differentiation process was examined for probing the underlying mechanism of IL-4-promoted myogenesis. While IL-4R was significantly down-regulated at early myogenic stage (2-4 day) but recovered to the comparable level to their parental cells (on day 0) at late stage (day 6), this differentiation-dependent pattern of IL-4R expression was abolished in cells with IL-4 exposure (Figure 2(c)). GATA3 were consistently expressed throughout the differentiation process, and IL-4 further augmented their expression. Additionally, IL-4 released by mature myocytes was increased about 6 folds compared to the parental myoblasts (Figure 2(d)). It suggests that C2C12 cells may secrete IL-4 to enhance myogenesis by autocrine and/or paracrine effects through its corresponding receptor and signaling pathway.

### 3.3. Effects of IL-4 on Insulin Signaling in Myoblasts

**Figure 3:**
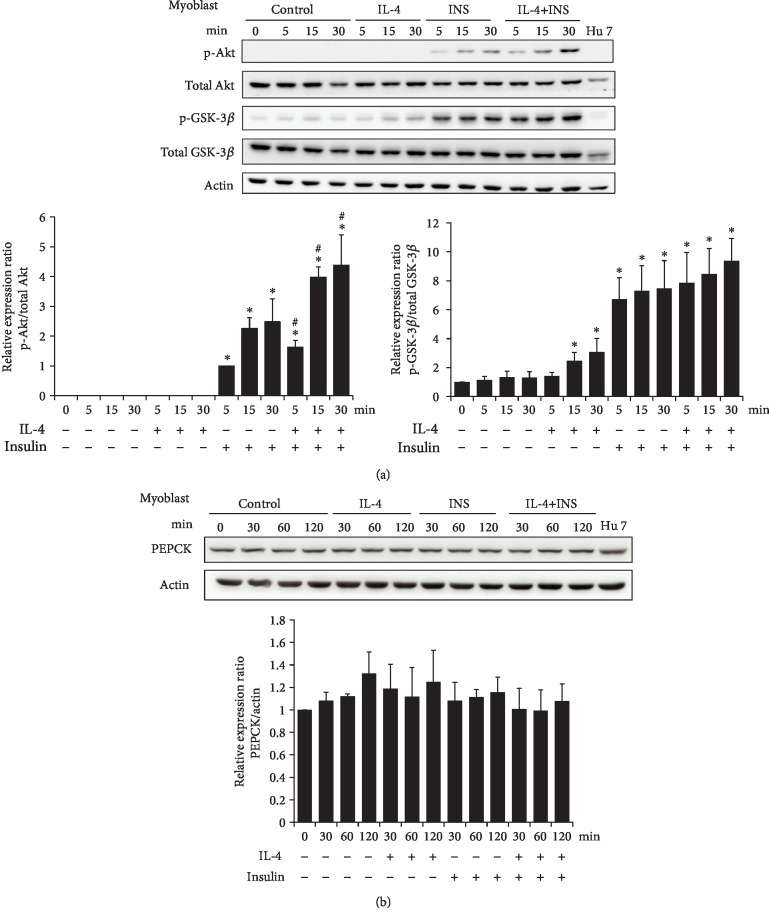
IL-4 boosts insulin signaling in C2C12 myoblasts. Cells were transferred to serum free medium for 3 hr, followed by exposure to IL-4 and/or insulin for 30 min. (a) Cell lysate were harvested at indicated time, and p-Akt and p-GSK-3*β* were analyzed (n = 3). (b) Cells were transferred to KRPH buffer for 3 hr, and cellular glucose uptake was measured as described in Methods (n = 3). ^∗^*p* < 0.05 v.s 0 min, ^#^*p* < 0.05 v.s INS group at the same time point.

The effects of IL-4 on insulin signaling were next examined in myoblasts. While IL-4 did not affect p-Akt, p-GSK-3*β* was significantly increased after 15 min of IL-4 treatment and insulin-induced p-Akt was enhanced by IL-4 (Figure 3(a)). While IL-4 increased the insulin-stimulated glucose uptake, this elevated effect did not reach statistical significance (data not shown). In addition, the levels of critical enzyme for gluconeogenesis, phosphoenolpyruvate carboxykinase (PEPCK) remained consistent in the presence of IL-4 and/or insulin treatment (Figure 3(b)). Therefore, IL-4 plays a synergistic role to insulin efficacy and tends to enhance insulin-stimulated glucose uptake in myoblasts.

### 3.4. Regulation of Myocyte GLUT4 Translocation and Glucose Uptake by IL-4

**Figure 4:**
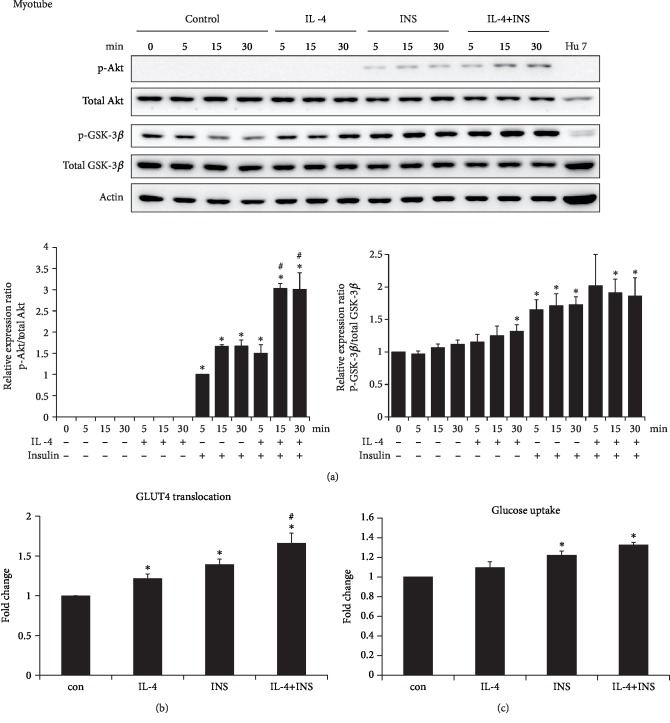
IL-4 promotes insulin signaling and GLUT4 translocation in C2C12 myocytes. Myocytes were transferred to serum free medium for 3 hr, followed by exposure to IL-4 and/or insulin for 30 min. (a) Cell lysate were harvested at indicated time, and levels of p-Akt and p-GSK-3*β* were analyzed (n = 3). ^∗^*p* < 0.05 v.s 0 min, ^#^*p* < 0.05 v.s INS group at the same time point. (b) GLUT4 translocation and (c) glucose uptake was assayed as described in Methods (n = 3). ^∗^p < 0.05 v.s control, ^#^p < 0.05 v.s INS.

Similar to the scenario in myoblasts, concomitant IL-4 treatment boosted insulin signaling by significantly increased insulin-induced p-Akt in mature myocytes (Figure 4(a)). IL-4 alone enhanced GLUT4 translocation, and insulin-stimulated GLUT4 translocation were further boosted by IL-4 (Figure 4(b)). Myocyte glucose uptake was not significantly enhanced in cells with IL-4 exposure, and a marginal significance regarding the insulin-induced glucose uptake in the present of concomitant IL-4 treatment was observed (Figure 4(c)). It indicates that IL-4 harbors synergistic effects by boosting insulin-induced GLUT4 translocation, and shows the tendency to elevate the insulin-stimulated glucose uptake. Collectively, IL-4 exerts positive regulatory capacity to glucose metabolic machinery and promotes insulin efficacy in myoblasts and mature myocytes.

### 3.5. In Vivo Effects of IL-4 on Muscle Cells

**Figure 5:**
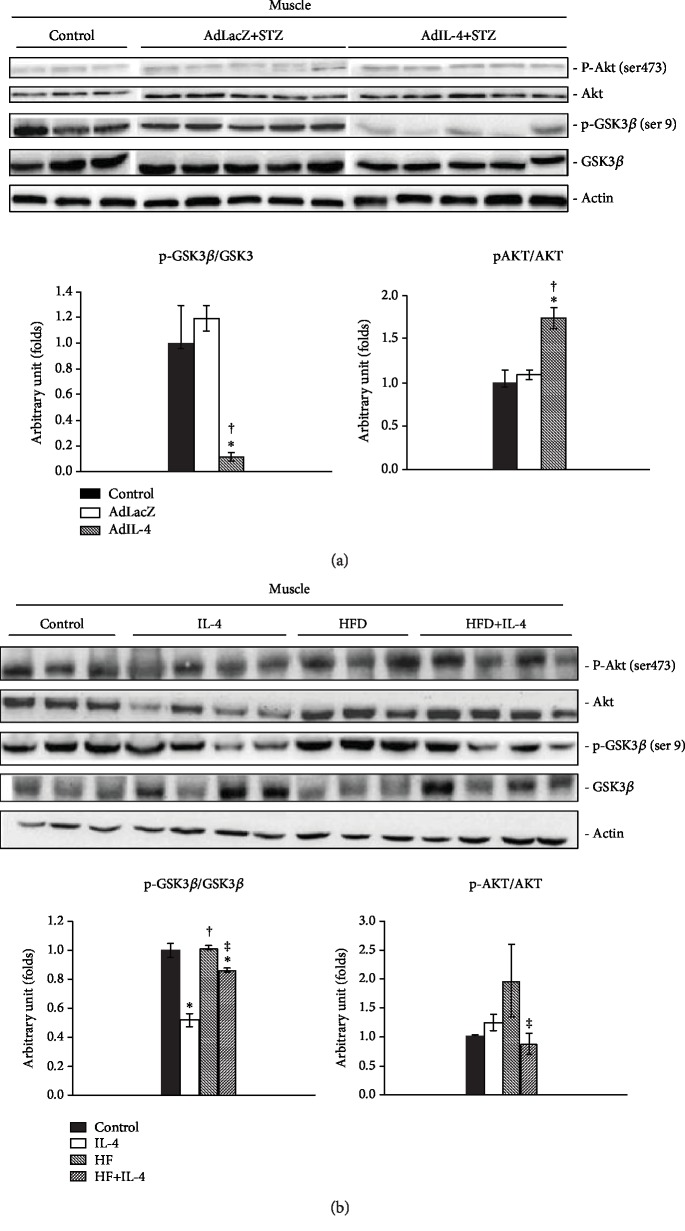
Regulation of Akt and GSK3*β* by IL-4 in muscle under insulin resistance and diabetic status. (a) Results of Western blotting showing the alternations of p-Akt and p-GSK-3*β* in quadriceps tissues obtained from STZ-induced diabetic mice administered with adenovirus containing full-length IL-4 (AdIL-4 mice) mice receiving IL-4 injection (HFD + IL-4 mice). ^∗^p < 0.05 v.s control, ^†^p < 0.05 v.s AdLacZ, n = 3-5. (b) Western blotting showing the alternations of p-Akt and p-GSK-3*β* in quadriceps tissues obtained from obesity-induced insulin resistant mice receiving IL-4 injection (HFD + IL-4 mice). ^∗^p < 0.05 v.s control,^†^p < 0.05 v.s IL-4, ^‡^p < 0.05 v.s HFD; n = 3-4.

The influences of IL-4 to Akt and GSK-3*β* in muscle cells under diabetic and insulin resistant status were subsequently examined *in vivo*. Primary muscle tissues were obtained from STZ-induced diabetic mice administered with adenovirus containing full-length IL-4 (AdIL-4 mice) to mimic diabetic condition with transient IL-4 overexpression [12]. Interestingly, p-GSK-3*β* was decreased while p-Akt was significantly increased in primary muscle tissues obtained from transient IL-4 overexpressed STZ-induced diabetic mice (AdIL-4 mice, Figure 5(a)). It echoes our previous finding [12] and suggests that the IL-4-improved glucose tolerance results from enhancing insulin action by up-regulating Akt activity in muscle.

Meanwhile, obesity-induced insulin resistant status with long-term IL-4 overexpression was also established by feeding IL-4-injected mice with HFD (HFD + IL-4 mice) [12]. Intriguingly, p-Akt was significantly decreased in muscle from HFD + IL-4 mice (Figure 5(b)) while p-GSK-3*β* was significantly decreased as in AdIL-4 mice (Figure 5(a)). The results suggest that although transient and long-term IL-4 overexpression show differential regulation to p-Akt, IL-4 down-regulates p-GSK-3*β* under insulin-resistant status. Transient IL-4 treatment boosts insulin signaling by enhancing p-Akt, which may result in improved glucose tolerance and insulin sensitivity [15]. On the contrary, long-term IL-4 treatment downregulates p-Akt and decreased insulin efficacy in muscle from HFD mice.

## 4. Discussion

IL-4 is a myoblast recruitment factor to promote morphological transition from myoblasts to mature myotubes [19]. Following the initial fusion of myoblasts to form myotubes, IL-4 treatment leads to further nuclear addition and increased myotube size. In support of the conclusion that IL-4 promotes muscle differentiation and regeneration [19, 20], this study demonstrates that IL-4-treated mature myotubes contain more nuclei with longer diameter (Figure 2(b)). In addition, IL-4 promotes myogenesis by enhancing the expression of myocyte marker genes, as well as myogenesis-promoting transcription factor and kinase at different stages (Figure 2(a)). The elevated IL-4 secreted from the mature myocytes (Figure 2(d)) suggests that IL-4 promotes myogenesis through autocrine and/or paracrine mechanisms. These observations not only reveal the networking between immune-regulatory system and metabolic organs, but also suggest that IL-4 is a potential adjuvant therapeutic target for sarcopenia via its activity of promoting myogenesis to preserve muscle mass.

Accumulating evidence indicates that chronic low-grade systemic inflammation is a contributing and promoting factor for sarcopenia [22, 23]. Originally designated as “cachectin”, TNF-*α* is the most notable one among the pro-inflammatory cytokines leading to muscle atrophy [24, 25]. TNF-*α* suppresses myocyte differentiation by activating NF-*κ*B during immobilization-induced decrease of muscle mass [26, 27]. IL-1*β*, IL-6, and other related cytokines are also potential mediators of muscle wasting or atrophy. Collectively, clinical syndromes of sarcopenia are considered to be the net outcome from the combinatorial effects of multiple pro-inflammatory cytokines [28]. On the contrary, IL-15 inhibits muscle protein breakdown and mitigates apoptotic DNA fragmentation by counteracting TNF-*α* signaling [29]. The imbalance between pro- and anti- inflammatory cytokines during aging or certain diseases is contributed and/or accelerates muscle atrophy and sarcopenia. Therefore, the anti-inflammatory cytokines are suggested to ameliorate muscle atrophy and sarcopenia by antagonizing the expression and/or activities of pro-inflammatory cytokines.

In this context, it is reasonable to suggest that IL-4 has potential to be an adjuvant therapeutic target for sarcopenia owing to its multiple activities. First of all, sarcopenia is suggested to be an early predictor for the susceptibility of insulin resistance, diabetes and metabolic syndrome, and vice versa [22]. Our previous reports uncover the association between IL-4 genotypes and diabetic susceptibility [10]. Therefore, it would be intriguing to characterize the IL-4 genotypes in subjects with sarcopenia. Secondly, type 2 innate immunity is required for skeletal muscle regeneration after injury [20]. Muscle damage rapidly recruits IL-4-secreting eosinophils to elicit the regenerative activity of muscle resident fibro/adipocyte progenitors (FAPs). IL-4 signaling promotes FAPs activity of clearing necrotic debris to support myogenesis while inhibits FAPs differentiation into adipocytes [20]. In addition, Akt activation completely protect against muscle injury caused by eccentric contraction [22]. Taking the findings of above study, our previous reports in which IL-4 suppresses adipocyte differentiation [14, 15] and the present study that IL-4 boosts insulin-induced Akt activation together, we speculate that IL-4 may suppress muscle atrophy-induced damage and promotes muscle regeneration via elevating Akt activity through its insulin-synergizing activity [12, 30, 31]. Thirdly, myokines from skeletal muscle are suggested to prevent insulin resistance by counteracting the adverse metabolic effect of adipokines [32]. Increased whole-body lean mass in elderly subjects with sarcopenia improves insulin sensitivity [32]. The elevated IL-4 secreted from the mature myocytes (Figure 2(d)) suggests that IL-4 may exhibit dual beneficial functions of promoting myogenesis and improving insulin sensitivity through autocrine and/or paracrine mechanisms. The attenuation of down-regulated IL-4R at early myogenic stage by IL-4 (Figure 2(c)) further supports the participation of IL-4 in autocrine regulatory circuit during myogenesis. Accordingly, our finding echoes that differentiating muscle cells and regenerating myofibers secrete IL-4 to stimulate myoblast fusion and muscle fiber growth [19]. Fourthly, as the scenario of IL-15 [33], it is very likely that IL-4 ameliorates muscle atrophy via inhibiting pro-inflammatory cytokines, especially IL-6 and TNF-*α*, on the basis that IL-4 almost entirely suppresses the expression and activities of these cytokines [34, 35]. The present study provides new clues suggesting IL-4 as a potential target to counteract the risk factors leading to muscle dystrophy.

Skeletal muscle is the largest insulin-targeting organ which uptakes more than half of the postprandial glucose and consumes approximately 20% of the body's energy [36]. Inefficient response of muscle cells to insulin is a major contributor to systemic insulin resistance and metabolic dysregulation. For addressing the role of IL-4 in muscle energy metabolism, the regulation and corresponding mechanism of IL-4 to glucose metabolism in myoblasts and muscle cells were analyzed. IL-4 does not cause significant changes of p-Akt and p-GSK-3*β*, nevertheless, it aids insulin signaling by boosting Akt activity (Figures 3(a) & 4(a)). Moreover, IL-4 promotes GLUT4 translocation in muscle cells and tends to boost the insulin-induced glucose uptake (Figures 4(b) & 4(c)). Our most recent observations demonstrate that the insulin-stimulated glucose uptake in insulin-targeting hepatocytes and adipocytes is consistently increased about 1.25-1.5 folds [18]. Therefore, IL-4 serves as a synergistic agent to insulin for promoting glucose uptake which, at least in part, accounts for our previous finding that IL-4 improves glucose tolerance and insulin sensitivity [12]. Although the IL-4-enhanced muscle glucose uptake does not reach statistical significance (Figure 4(c)), the enhanced GLUT4 translocation and the total amount of glucose entering muscle cells under IL-4 exposure should not be overlooked since skeletal muscle is the largest insulin-targeting organ which accounts most of the body energy expenditure.

Alterations of p-Akt and p-GSK-3*β* under transient or long-term IL-4 administration were examined to explore the *in vivo* effects of IL-4 on muscle cells with diabetic and insulin resistant conditions. While transient IL-4 augments insulin signaling (AdIL-4 mice, Figure 5(a)), p-Akt is significantly decreased in mice receiving long-term IL-4 (HFD + IL-4 mice, Figure 5(b)). Nevertheless, both short- and long- term IL-4 significantly reduced p-GSK-3*β*. The results indicate that IL-4 is insufficient to inhibit GSK-3*β* activity under insulin resistant and diabetic conditions. The incompetency of long-term IL-4 injection to ameliorate insulin resistance is probably due to physical adaptation and/or compensation under consistent IL-4 exposure for meeting the *in vivo* energy requirements to maintain homeostasis [37, 38].

It is intriguing that *in vivo* and *in vitro* IL-4 treatments exert differential effects on regulating GSK-3*β* in muscle. Besides, *in vivo* short- and long- term IL-4 exhibit opposite regulation to Akt. In particular, IL-4 promotes glucose tolerance through activating Akt while suppressing GSK-3*β*. The above observations may be explained by the following perspectives. First of all, liver, instead of the energy-consuming muscle, is the major reservoir of glycogen. Postprandial insulin signaling inhibits hepatic GSK-3*β* phosphorylation, which in turn results in glycogen synthase activation and glycogen synthesis. Fibroblast growth factor 19 is reported to facilitate postprandial glycogen synthesis through insulin-independent pathway [39]. In this context, IL-4 might display differential regulatory function to different insulin-targeted organs. As a matter of fact, a recent study provides evidence concerning differential modulation of white adipose and hepatic energy metabolism by plasma exosomal miRNA [40]. Secondly, although C2C12 myocyte is an ideal model for studying molecular mechanism, *in vitro* results may not faithfully reflect the net outcome of the inter-organ network between certain cells/organs and their surrounding microenvironments. The results from cell study suggest that IL-4 synergistically enhances insulin efficacy under physiological insulin-sensitive condition. Nevertheless, transient IL-4 boosts insulin signaling through activating Akt while long-term IL-4 down-regulates Akt activity in diabetic and insulin-resistant conditions. Both transient and long-term IL-4 treatments inhibit glycogen synthesis. It suggests that insulin sensitivity determines the response of muscle cells to environmental stimuli. Therefore, the inefficiency of long-term IL-4 to enhance Akt activity might be due to *in vivo* adaptation for counteracting metabolic alterations caused by the consistent elevated cytokines. Thirdly, the experimental design, including the doses and period of exposure to certain stimulus, would affect the results in *in vitro* and *in vivo* environments. Study from Szekeres, et al. also documented conflicting *in vivo* and *in vitro* results concerning the regulation of skeletal muscle glucose metabolism by TBC1D1 [41], the Akt substrate Rab-GTPase-activating protein. The authors demonstrate that basal and insulin-induced GLUT4 translocation is increased in *Tbc1d1*-silencing L6 muscle cells. However, insulin-stimulated glucose uptake in glycolytic muscle harvested from TBC1D1-deficient mice was enhanced *in vivo* but profoundly impaired *in vitro*. The exact reasons contributing to and explaining these contradictory effects remains unidentified. The authors assumed that the alterations of certain *in vivo* skeletal muscle-interacting factors in TBC1D1-deficient mice, such as the circulating factors, hormones or nutrient supply, may lead to the paradoxical observations. In this context, we speculate this may be another possible molecular mechanism underlying our conflicting findings since IL-4 mediates muscle cell behaviors through the major insulin signaling molecule Akt which subsequently modulates TBC1D1 activity.

Taken our results together, the net effect of IL-4 on muscle under physiological condition is to promote catabolism for meeting the energy needs by enhancing glucose uptake. Therefore, better glucose tolerance and insulin sensitivity are achieved. Nevertheless, insulin resistance takes the dominant role in muscle cells responding to IL-4 for maintaining homeostasis and avoiding cellular apoptosis or damage [42].

T2DM is associated with chronic inflammation with elevated levels of pro-inflammatory cytokines [42, 43]. IL-4 modulates expression of pro-inflammatory cytokines and inflammation mediators [43]. In animal models, IL-4 production from diet-induced obese mice is increased [44] and serum IL-4 is decreased in Sprague-Dawley rat after receiving visceral fat remove surgery [45]. In human study, T2DM patients carry higher IL-4 secreting genotypes [10]. IL-4 secreted from adipocytes and hepatocytes exerts the capacity of modulating local immune response and insulin sensitivity [42, 46]. These results suggest that IL-4 participates in diet-induce obesity and metabolism. Combining the conclusions from our study [10–15], IL-4 promotes glucose tolerance and insulin sensitivity through boosting insulin signaling by altering Akt and GSK-3*β* activities and counteracting the effects of pro-inflammatory cytokines. In addition, IL-4 is involved in lipid metabolism by inhibiting adipogenesis and promoting lipolysis [47]. IL-4 is also characterized to promote insulin signaling in hepatocytes [18], and insulin signaling is impaired in mice with deficiency of IL-4 downstream STAT6 [48]. Collectively, the overall effect of IL-4 is to promote catabolism under physiological condition. Nevertheless, under insulin-resistant status, especially the environment of extensive elevated IL-4, physical adaptation would take over to counteract the metabolic alterations caused by consistently elevated cytokine exposure for maintaining energy homeostasis.

In addition to the wide-recognized anti-inflammatory activity, IL-4 modulates myogenesis and energy metabolism through multiple functions. IL-4 promotes glucose tolerance and insulin sensitivity through activating Akt and GSK-3*β* [12]. In adipocytes, IL-4 inhibits lipogenesis and promotes lipolysis to decrease lipid deposits [14, 15, 47]. In hepatocytes, IL-4 boosts insulin-induced energy deposits by enhancing glucose uptake and lipogenesis [18]. In the present study, we reveal that IL-4 promotes myogenesis and improves glucose metabolic efficacy in muscle cells, which further suggest that IL-4 has dual potential to act as an adjuvant therapeutic target for sarcopenia and insulin resistance. This study provides evidence to address the roles of cytokine in mediating whole-body muscle reservoir and glucose metabolism, as well as the interaction between immune responses and energy homeostasis, which implicates the regulation of immune system to the muscle differentiation and exercise performance.

## Figures and Tables

**Table 1:** Primers sequences used in the study.

Target gene	Sequence (5'-3')
MyHC-F	AGGCGGCTGAGGAGCACGTA
MyHC -R	GCGGCACAAGCAGCGTTGG
Myogenin-F	GATTGTGGGCGTCTGTAGGGT
Myogenin-R	GAAGCGCAGGCTCAAGAAAGT
MURC-F	ACAGTCACACAGCAATACGGGCTA
MURC-R	AAGAGTCTGCCGTGTCTTCTGCAT
18 s-F	CGCCGCTAGAGGTGAAATTCT
18 s-R	CATTCTTGGACCCTGCTTTCG

## Data Availability

Data used to support the findings of this study are included within the article.

## Abbreviations

2-NBDG: 2-[N-(7-nitrobenz-2-oxa-1,3-diazol-4-yl)amino] -2-deoxy-D-glucose
AdIL-4: adenovirus containing full-length IL-4
CDK5: cyclin-dependent kinase 5
GATA3: GATA-binding protein 3
GLUT4: glucose transporter 4
GSK-3: glycogen synthase kinase 3
HDL-C: high density lipoprotein-cholesterol
HFD: high fat diet
HS: horse serum
IHC: immunohistochemistry
IL: interleukin
IL-4R*α*: interleukin-4 receptor *α* chain
INS: insulin
MURC: muscle-restricted coiled-coil protein
MYF6: myogenic factor 6
MyHC: myosin heavy chain
PEPCK: phosphoenolpyruvate carboxykinase
p-: phosphorylated-
SD: standard chow diet
SF: serum free/serum starvation
STZ: streptozotocin
T2DM: type 2 diabetes mellitus
TNF-*α*: tumor necrosis factor-*α.*
